# 

**Published:** 1915-06

**Authors:** 


### Local Medical Notes.

**Royal Mineral Water Hospital, Bath.**—The number of invalided soldiers treated in this hospital are: British soldiers, 301; Belgian soldiers, 110.

The first case was admitted on September 28th.

The hospital was offered to the War Office for the reception of sick and wounded, but was accepted for such cases only as require the bath treatment.

Cases admitted include fibrositis, arthritis, rheumatism, rheumatic myositis, sciatica, frost-bite, muscular wasting due to wound, neurasthenia, muscular strain, and the after-effects of wounds.

The number of beds is 150. The average number of soldiers recently over 100.

Massage has been largely used and with success, also ionisation.

**Sanatorium Work in Bristol.**—For the following report we have to thank our Medical Officer of Health, Dr. D. S. Davies.

The complete Tuberculosis Scheme for the City of Bristol adopted by the Council on 22nd July, 1913, included—

*A. Dispensaries—Two.* (*a*) 19 Portland Square.
(*b*) 4 Redcliffe Parade West.

*B. Sanatoria and Hospitals.*
(*a*) Sanatoria. (1) Winsley—50 beds for early cases.
(2) Ham Green—40 beds.[1]
(*b*) Hospitals. Ham Green—20 beds.[1]

The Scheme was to be administered under the general administrative supervision of the Medical Officer of Health by—

1. Tuberculosis Officer, who would assume general clinical control of the City Dispensaries and of all Tuberculosis work in the City, and would act as adviser in Tuberculosis work of the Insurance Committee.

2. Resident Medical Officer at Ham Green, in clinical charge of the Hospital and Sanatorium at Ham Green.

3. An Assistant Medical Officer for Sanatorium and Dispensary work.

In the early part of 1914 the Scheme had considerably advanced :—

*A. Dispensaries.*

The Redcliffe Dispensary was the first to be opened, having been taken over from the Civic League in January, 1913. During the two years 1912–13 the number of new patients seen at this Dispensary was 1,581, and the number of attendances of patients amounted to 14,861.

The Portland Square Dispensary, opened in May, 1914, is larger and more completely equipped than the original home, and includes a pharmacy, a laboratory and three observation beds for special cases, also accommodation for a nurse, as well as the usual waiting and consulting rooms.

### Medical Staff.

Considerable dislocation of work has been caused by the outbreak of war in August. The Tuberculosis Officer (Dr. Faill) and the Assistant Medical Officer (Dr. Galpin) have both been called away on active service, and Dr. Hill is now Acting

[1] For all classes of suitable cases and after-care cases.

Tuberculosis Officer, with Dr. Penny as Assistant Medical Officer.

With the opening of the Portland Square Dispensary two extra nurses were added to the staff, making a total of four.

One of the Tuberculosis nurses has also left on war service, necessitating the making of a temporary appointment.

The number of new cases attending the Dispensaries during the year 1914 was 991, a considerable increase on the 696 during the year 1913. The subsequent visits during the year numbered 9,850.

*B. Sanatoria.*

Until the completion of the Sanatorium and Hospital beds now in course of erection at Ham Green, the accommodation available will not amount to the full figures of the Scheme.

At present there are available and in use at

| | |
|---|---|
| Winsley .. .. .. | 50 |
| Ham Green .. .. .. | 20 |
| Clift House (advanced cases) | 28 |

The Ham Green Sanatorium beds are provided in temporary shelters, viz. :—

| | | | |
|---|---|---|---|
| One .. .. .. .. | for | 16 | patients. |
| One .. .. .. .. | for | 9 | ,, |
| Seven single shelters .. .. | for | 7 | ,, |
| Part of permanent Fever Block | for | 8 | ,, |
| | | 40 | |

but at present (May, 1915), owing to the fever necessities of war time, the number accommodated has had to be limited to 20, and when the first part of the new Sanatorium buildings are completed these will have to be temporarily devoted to the same service.

Clift House Hospital has afforded useful temporary accommodation for 28 advanced cases, but these premises will shortly be demolished for Dock purposes, and by that time it is hoped that the Hospital buildings for late cases at Ham Green will be available for use.

The number of Sanatorium and Hospital cases dealt with during the year 1914 are here shown :—

| | Male. | Female. | Total. |
|---|---|---|---|
| Winsley .. .. | 85 | 41 | 126 |
| Ham Green .. .. | 29 | 73 | 102 |
| Clift House .. .. | 44 | 51 | 95 |
| | 158 | 165 | 323 |

